# Tender leaf and fully-expanded leaf exhibited distinct cuticle structure and wax lipid composition in *Camellia sinensis cv Fuyun 6*

**DOI:** 10.1038/s41598-018-33344-8

**Published:** 2018-10-08

**Authors:** Xiaofang Zhu, Yi Zhang, Zhenghua Du, Xiaobing Chen, Xin Zhou, Xiangrui Kong, Weijiang Sun, Zijian Chen, Changsong Chen, Mingjie Chen

**Affiliations:** 1 College of Horticulture and Fujian Provincial Key Laboratory of Haixia Applied Plant Systems Biology, Fujian Agriculture and Forestry University, Fuzhou, Fujian 350002 China; 2 Tea Research Institute, Fujian Academy of Agricultural Sciences, Fuan, Fujian 355000 China; 3 FAFU-UCR Joint Center/Horticultural Plant Biology and Metabolomics Center, Haixia Institute of Science and Technology, Fujian Agriculture and Forestry University, Fuzhou, Fujian 350002 China; 4 Anxi College of Tea Science, Fujian Agriculture and Forestry University, Fuzhou, Fujian 350002 China; 5 Engineer School, University of Missouri, Columbia, Missouri 65211 USA

## Abstract

The goal of the present study was to compare the structural and compositional differences of cuticle between tender leaf and fully-expanded leaf in *Camellia sinensis*, and provide metabolic base for the further characterization of wax biosynthesis in this economically important crop species. The tender second leaf and the fully-expanded fifth leaf from new twig were demonstrated to represent two different developmental stages, their cuticle thickness were measured by transmission electron microscopy. The thickness of the adaxial cuticle on the second and fifth leaf was 1.15 µm and 2.48 µm, respectively; the thickness of the abaxial cuticle on the second and fifth leaf was 0.47 µm and 1.05 µm, respectively. The thickness of the epicuticular wax layer from different leaf position or different sides of same leaf were similar. However, the intracuticular wax layer of the fifth leaf was much thicker than that of the second leaf. Total wax lipids were isolated from the second leaf and the fifth leaf, respectively. Gas chromatography-mass spectrometry analysis identified 51 wax constituents belonging to 13 chemical classes, including esters, glycols, terpenoids, fatty acids and their derivatives. Wax coverage on the second and fifth leaf was 4.76 µg/cm^2^ and 15.38 µg/cm^2^, respectively. Primary alcohols dominated in the tender second leaf. However, triterpenoids were the major components from the fully-expanded fifth leaf. The predominant carbon chains varied depending on chemical class. These data showed that the wax profiles of *Camellia sinensis* leaves are development stage dependent, suggesting distinct developmental dependent metabolic pathways and regulatory mechanisms.

## Introduction

The plant cuticle covers almost all terrestrial plants, and plays multiple roles in the interaction between plants and the environment. It reduces non-stomatal water loss^1^, protects plants from ultraviolet radiation^2^, minimizes pollutant retention on leaf surfaces^3,4^, defends against bacterial and fungal pathogens^5^, participates in plant-insect interaction^6^, regulates pollen-pistil interaction^7^, and prevents organ fusion^8–10^. These diverse functions are realized through the physical and chemical properties of cuticular waxes, thus a large volume of research had been dedicated to characterize cuticular waxes composition in different plant species^11–17^. These investigations established that plant cuticle waxes are predominantly comprised of very long chain fatty acids and their derivatives such as aldehydes, alkanes, branched alkanes, primary alcohols, secondary alcohols, unsaturated fatty alcohols, ketones, and wax esters, as well as cyclic compounds including triterpenoids and sterols^18–20^.

It is widely accepted that cuticular wax composition varies both qualitatively and quantitatively between plant species, between organs or even tissues of the same species. For the same type of tissue within same species, wax composition also changes during development. Tulloch (1973) found that formation of wax constituents on species of *Triticum* varied with age^21^, octacosanol being formed preferentially during the early stages of growth of *T. durum* whereas the production of β-diketones increased as the leaves expanded. Baker (1979) reported that the relative proportions of the constituent classes of *Prunus persica* leaf waxes altered steadily during development^22^, increased in alkyl ester production on the adaxial surface and hydrocarbons on the abaxial surface compensating for the marked reduction in triterpenoid synthesis. Freeman (1979) also proved that the composition of the leaf and fruit waxes of *Citrus* spp. also varied with age of the tissue^23^. They found that secondary alcohols, dominant in the post-emergent leaf wax, declined rapidly during the remaining stages of expansion and were replaced by primary alcohols and hydrocarbons. Contrastingly, as primary alcohol and hydrocarbon production decreased during development of Citrus fruits, synthesis of aldehydes and fatty acids increased, such that these constituents became the major components of the ripe fruits. Jetter and Schäffer (2001) developed a new method to isolate epicuticular waxes from *Prunus laurocerasus* leaf, then did real time course analyses to monitor cuticle changes during leaf development^11^. They found that alkyl acetates accumulated during epidermal cell expansion, and then alcohols dominated, followed by alkane accumulation. In contrast, the intracuticular waxes of the *Prunus laurocerasus* leaf stayed fairly constant during development. For *Kalanchoe daigremontiana*, leaf waxes coverage increased steadily, triterpenoids dominated the wax mixture throughout leaf development, but decreased in mature leaves, while VLCFA derivatives increased^15^. Shumborski *et al*.^24^ characterized cuticle changes during Arabidopsis stem development by comparing the rapidly expanding young stems and fully elongated older stems, and revealed fine structural changes of the stem cuticle during stem development^24^. These studies suggested that cuticle changes during development are likely species-specific.

Tea is the oldest non-alcoholic caffeine-containing beverage in the world, and is made from the tender leaves of tea tree (*Camellia sinensis* L.). Postharvest withering is an essential step for manufacture of several types of tea including black tea, Oolong tea or white tea^25^. However, not much attention was paid to study how cuticle affects the processing properties of the postharvested tea leaf. Recently, some potent bioactive compounds such as policosanol (PC) was identified from green tea^26^. PC is a trivial name of a mixture of long-chain (C20-C36) aliphatic primary alcohols, its storage sites in fresh tea leaves remains unknown. It has long been noticed that the aroma quality of black tea and oolong tea is positively associated with leaf cuticle^27,28^, raising the possibility that some aroma precursors could be stored in leaf cuticular waxes. However, so far there is no comprehensive documentation of the tea leaf cuticle, it also remains unknown how they change with leaf growth. Tea tree also is an excellent system for cuticle research: (1) it is perennial evergreen shrub, well adapted to growth chamber or green house conditions; new twigs can emerge year around, thus facilitate obtaining research material; (2) tea tree can be easily propagated by short nod cutting, large materials with same genetic background can be easily obtained; (3) tea tree is self-incompatible, the out-crossing generated huge genetic variation, thousands of germplasms have been systemically collected in China. It is expected that there are large variations in cuticle structure and composition, this would provide new opportunities to discover new pathways for wax lipid biosynthesis, or perform association studies among cuticle structure, composition and water sealing property. In this study, we compared the cuticle and cuticular wax from the tender second leaf and the fully-expanded fifth leaf, we found that VLCFAs and their derivatives dominant in the cuticular waxes of the tender second leaf, while triterpenoids and steroids were the major components in the cuticular waxes of the fully-expanded leaf. This work provided metabolic base for the further characterization of cuticular wax biosynthesis in *Camellia sinensis*.

## Results

### Tea tree leaf expansion after bud break

**Figure 1:**
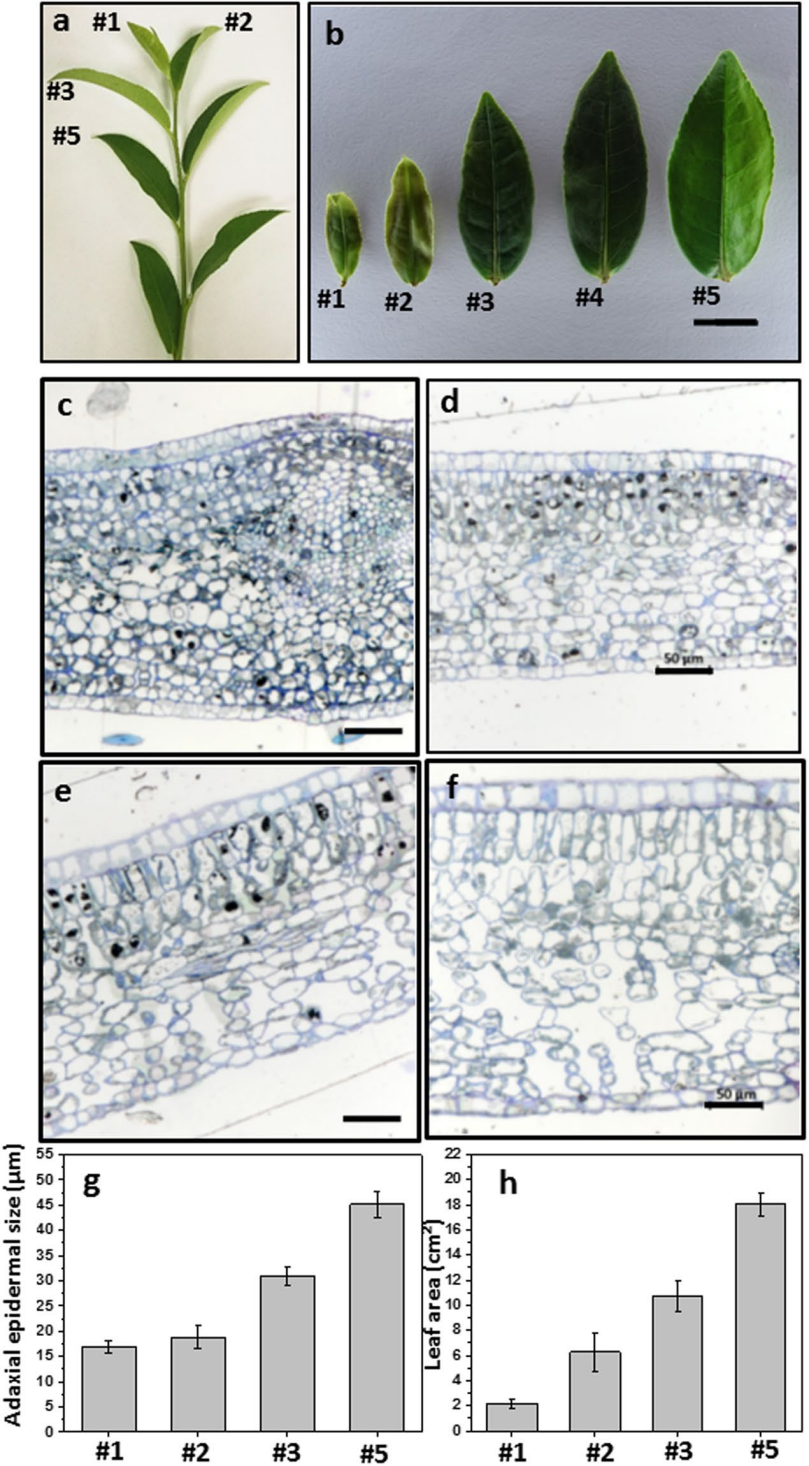
Different leaf positions from growing twig showed different cell size and structural characteristics in *Camellia sinensis cv Fuyun 6*. (**a**) Leaf positions from growing twig; (2) Morphological changes from the first leaf to the fifth leaf from same twig, bar = 1 cm; (**c–f**) Cross section of the first leaf (**c**), the second leaf (**d**), the third leaf (**e**) and the fifth leaf (**f**), bar = 50 μm; (**g**) Adaxial epidermal cell size at different leaf positions; (**h**) Adaxial surface leaf area at different leaf positions (n = 8). #1, the first leaf position; #2, the second leaf position; #3, the third leaf position; #5, the fifth leaf position.

Tea tree (*Camellia sinensis*) is an evergreen, perennial shrub or tree, characterized by more or less virgate stems^29^. Following bud break new leaves emerged from bud sequentially. Figure 1a showed a typical twig after bud break for 5 weeks, the second leaf and the fifth leaf were 10- and 28-day after bud break, respectively. After bud break leaf continued to expand (Fig. 1b). Cross sections were prepared from different leaf positions, we observed that the epidermal cell size of the first leaf and the second leaf remained constant (Fig. 1c,d), then become larger starting from the third leaf position (Fig. 1e,f). The epidermal cell sizes from different leaf positions were measured and the data was shown in Fig. 1g, which further confirmed our observation. Even though the epidermal cell size from the first leaf and the second leaf kept constant (Fig. 1g), the second leaf area was larger than the first leaf (Fig. 1h). These observations suggested that there was active cell division in the second leaf. Starting from the fourth leaf the increase in leaf length was slowed down, the leaf width continued to increase in the fifth leaf (Fig. 1b). The structural characteristics also were compared among different leaf position. In the first leaf all mesophyll cells showed round shape, no clear palisad layer formation, cells were tightly packed, no visible intercellular air space (Fig. 1c). In the second leaf palisad layer started to emerge, spongy layer became visible, cells were generally packed, only small intercellular air spaces were visible (Fig. 1d). In contrast, the third leaf and the fifth leaf showed all characteristics of a mature leaf: long cylindrical cells were arranged regularly in two rows and formed the palisad layer, the cells of the spongy layer were elongated and loosely packed, large intercellular air spaces clearly showed up (Fig. 1e,f). These cellular features indicated that the second leaf was tender, and the fifth leaf was fully-expanded.

### Cuticle thickness and ultrastructure determined by transmission electron microscopy

**Figure 2:**
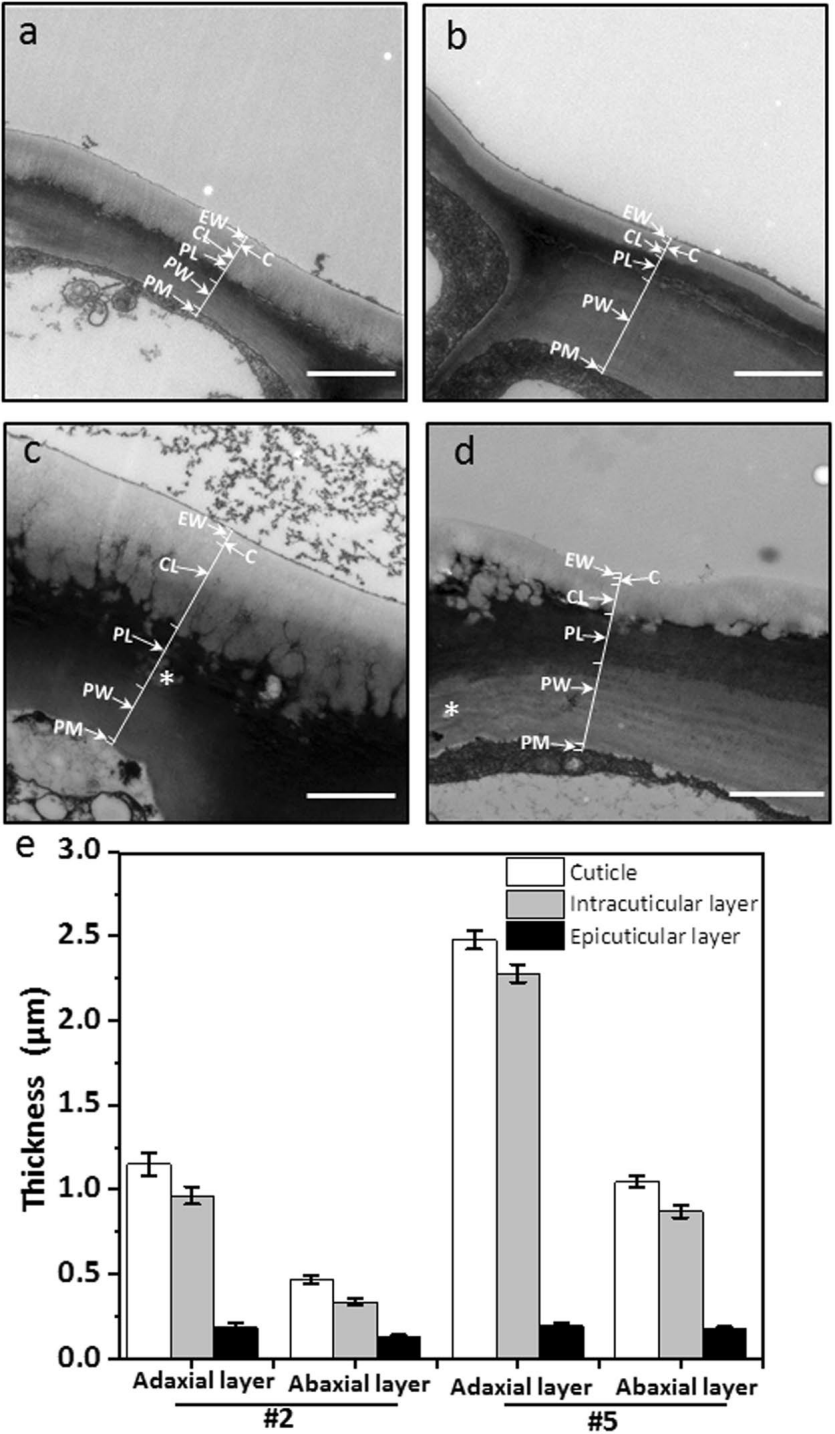
TEM observation of cuticle structure from the second leaf and the fifth leaf in *Camellia sinensis* cv ‘*Fuyun 6*’. (**a**) Adaxial side of the second leaf; (**b**) Abaxial side of the second leaf; (**c**) Adaxial side of the fifth leaf; (**d**) Abaxial side of the fifth leaf; bar = 2.0 μm. (**e**) The thickness of the leaf cuticle. #2, the second leaf position; #5, the fifth leaf position. Lipid inclusion bodies (indicated by asteroids^*^) were observed to present inside the cell wall. Epicuticular waxes (EWs) cover the cuticle proper (C), which is cutin embedded with intracuticular waxes (IWs). The cuticular layer (CL) seem also contain intracuticular wax. Pectinaceous layer (pL), primary cell walls (PWs) and the plasma membrane (PM).

To observe the structural differences of the cuticle between tender leaf and fully-expanded leaf, the second and the fifth leaf were fixed, cross sections were examined under transmission electron microscopy (TEM), and cuticle thickness from adaxial and abaxial surfaces were measured. Under TEM cuticle showed a whitish appearance covering the leaf epidermal cell layer. Cuticle proper formed a slightly electron-dense band, thus divided cuticle into epicuticular wax layer and cuticular layer (Fig. 2a–d)^30^. The cuticle thickness measurement indicated that the average total cuticle thickness from adaxial surface of the second leaf was 1.15 µm, the epicuticular wax layer and intracuticular wax layer was 0.19 µm and 0.97 µm, respectively; the average total cuticle thickness from abaxial surface of the second leaf were 0.47 µm, the epicuticular wax layer and intracuticular wax layer is 0.13 µm and 0.34 µm, respectively (Fig. 2a,b,e). In contrast, the average total cuticle thickness from adaxial and abaxial surfaces of the fifth leaf was doubled, and reached to 2.48 μm and 1.05 μm, respectively. On the adaxial surface of the fifth leaf the epicuticular wax layer and intracuticular wax layer was 0.20 μm and 2.28 μm, respectively; on the abaxial surface the epicuticular wax layer and intracuticular wax layer was 0.18 μm and 0.87 μm, respectively (Fig. 2e). These observations indicated that the cuticle on the adaxial surface is more than one-fold thicker compared to the cuticle on the abaxial surface of the same leaf, this difference was largely attributed to the intracuticular wax layer. The thickness for epicuticular wax layer from different leaf position only showed minor difference. In contrast, the thickness for intracuticular wax layer increased significantly with leaf growth (Fig. 2e). We also noticed that the epicuticular layer showed relatively straight outlines on the outer surface, which is true regardless of leaf position or leaf surface. However, for the intracuticular wax layer facing the cell wall side, the second leaf maintained relatively straight line. With leaf growth the original straight line was lost, and replaced by many small ridges, channels were observed between ridges (Fig. 2c,d). These structural alterations were less pronounced on the abaxial surface (Fig. 2b,d).

### Overall cuticle qualitative composition of the tender leaf and the fully-expanded leaf

**Figure 3:**
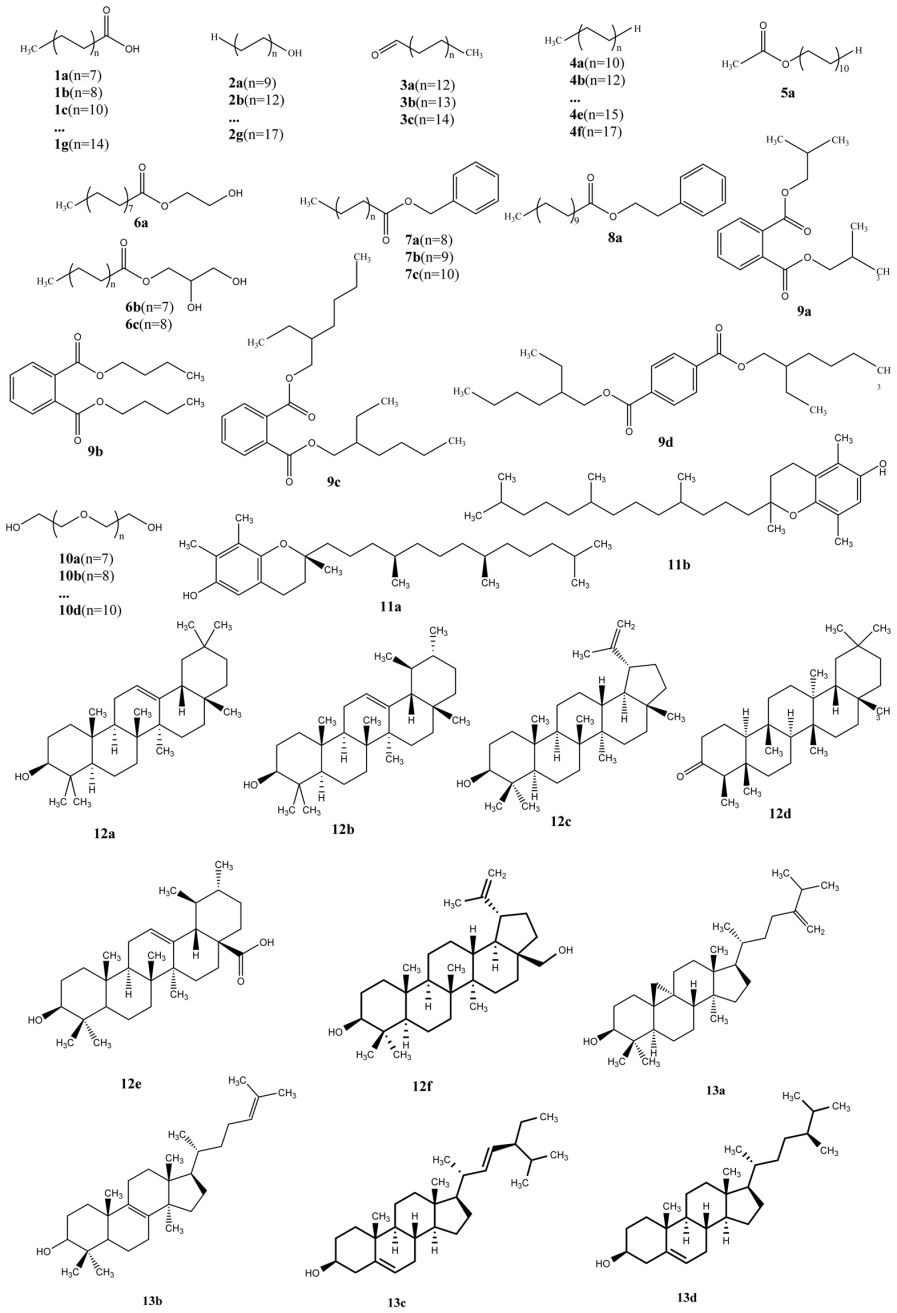
Compounds identified from the total wax mixtures of *Camellia sinensis* cv ‘*Fuyun 6*’.

To study possible cuticle compositional changes with leaf growth, waxes were sampled from whole leaf (the adaxial and abaxial surfaces) for chemical analyses by gas chromatography (GC)-flame ionization detector (FID) and mass spectrometry (MS). Preliminary screening revealed 40 and 42 different compounds from the wax mixtures of the second and the fifth leaf, respectively. Among the identified compounds 31 of them were commonly detected from both leaves. In total 51 different compounds were identified; 9 and 11 compounds were only detected from the second leaf and the fifth leaf, respectively (Supplementary Table S1). In addition, 11 and 16 unidentified compounds were present in the second leaf and the fifth leaf, respectively. Based on chemical structures, the 51 identified compounds were classified into 13 chemical classes (Fig. 3). Four groups, including free fatty acids, aldehydes, n-alkanes and 1-alkanols, were found commonly in the waxes of many species. The esters include 5 compound classes, and they differed in the combination of acyl moieties with various aliphatic or aromatic alcohols. Glycols were detected from both leaves. Finally, several compounds found only in the fifth leaf were identified as terpenoids.

### Total wax coverage between the second leaf and the fifth leaf

**Figure 4:**
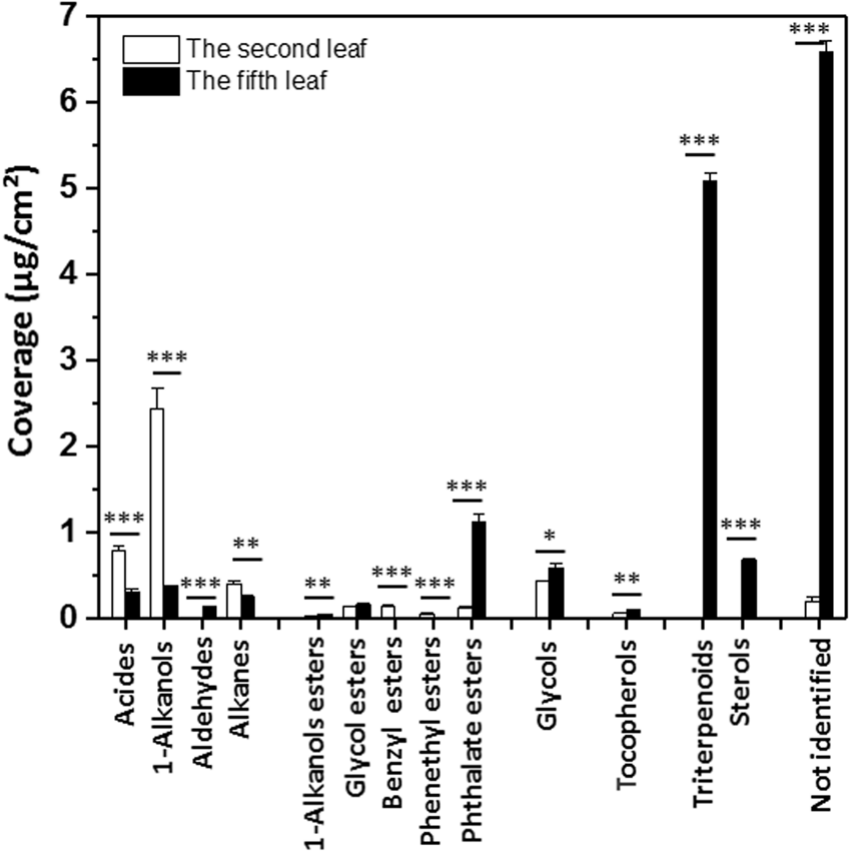
Compound class compositions of total wax mixtures of *Camellia sinensis* cv ‘*Fuyun 6*’. Coverages (μg/cm^2^) of compound classes within wax mixtures covering the second leaf and the fifth leaf. Bars represent mean ± standard error (n = 4). ^*^p < 0.05; ^**^p < 0.01; ^***^p < 0.001.

While 61% (31 out of 51) of the compounds were commonly detected from both leaf wax mixtures, they differed drastically in the relative proportions of these constituents. The mixture extracted from the second leaf was dominated by 1-alkanols (Figs 2a–g and 3), representing 51.21% of the GC-detected compounds (2.437 ± 0.235 μg/cm^2^) (Fig. 4). They were accompanied by substantial amounts of acids 1a–g (16.57%; 0.79 ± 0.05 μg/cm^2^). The compounds accounted to 9.0–2.0% of the total leaf wax mixtures included: glycols 10a-10d (8.87%; 0.422 ± 0.007 μg/cm^2^), alkanes 4a–f (8.20%; 0.390 ± 0.037 μg/cm^2^); benzyl esters 7a–c (2.89%; 0.137 ± 0.003 μg/cm^2^), phthalate esters 9a,b (2.43%; 0.115 ± 0.007 μg/cm^2^), glycol esters 6a/monoacyl glycerides 6b,c (2.76%; 0.131 ± 0.002 μg/cm^2^). Relatively small fractions of tocopherol 11a (1.05%; 0.050 ± 0.007 μg/cm^2^), phenethyl ester 8a (1.01%; 0.048 ± 0.002 μg/cm^2^), 1-alkanol ester 5a (0.32%; 0.015 ± 0.001 μg/cm^2^), aldehydes 3a,c (0.21%; 0.010 ± 0.0004 μg/cm^2^) and sterol 13c (0.15%, 0.007 ± 0.0004 μg/cm^2^) were also detected (Fig. 4). Taken together, the second leaf had a total wax coverage of 4.76 μg/cm^2^, including all identified and unidentified compounds. The identified compounds were accounted for 96% of the wax mass detected by GC.

In contrast to the second leaf, the wax mixtures of the fifth leaf were dominated by triterpenoids 12a–f (33.06%; 5.085 ± 0.100 μg/cm^2^), followed by phthalate esters 9a–d (7.27%; 1.119 ± 0.081 μg/cm^2^) (Fig. 4). Steroids 13a–e (4.36%; 0.671 ± 0.022 μg/cm^2^), glycols 10a–d (3.76%; 0.579 ± 0.052 μg/cm^2^), 1-alkanols 2a–d,f (2.39%; 0.368 ± 0.010 μg/cm^2^), acids 1a,b,d–f (1.95%; 0.301 ± 0.026 μg/cm^2^), and alkanes 4a–f (1.61%; 0.247 ± 0.007 μg/cm^2^) accounted for 6.0% to 1.0%, respectively. Some compounds were present in trace amount, including glycol esters 6a/monoacyl glycerides 6b,c (0.99%; 0.152 ± 0.012 μg/cm^2^), aldehydes 3a–c (0.80%; 0.123 ± 0.016 μg/cm^2^), and 1-alkanol esters 5a (0.27%; 0.041 ± 0.006 μg/cm^2^) (Fig. 4). In total, the fifth leaf had a wax coverage of 15.38 μg/cm^2^, and 42.91% of the wax mass detected by GC were unidentified compounds.

### Chain length distributions of common wax compound classes

**Figure 5:**
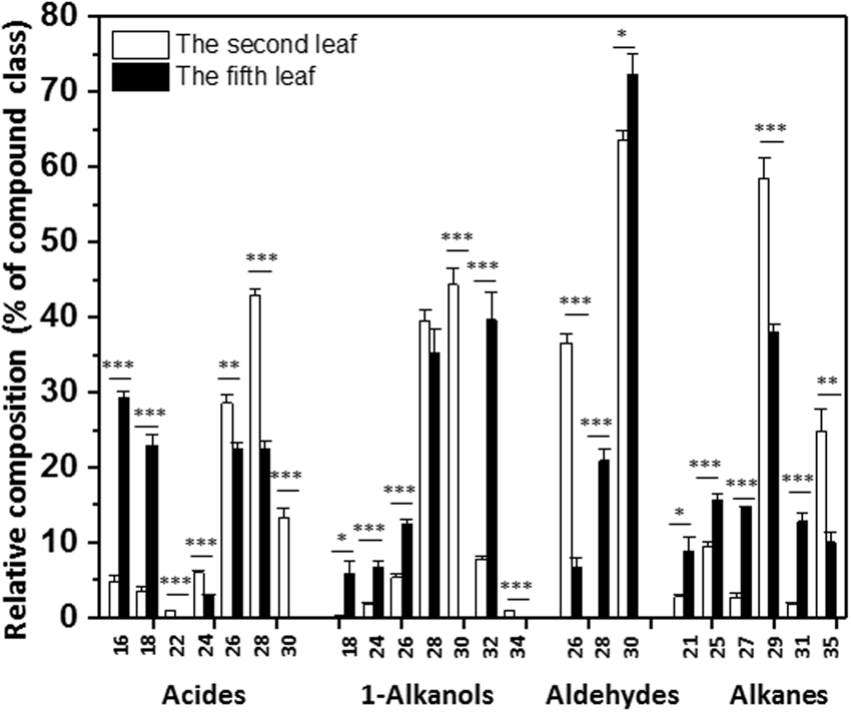
Chain length distributions of fatty acids and their derivatives in *Camellia sinensis* cv ‘*Fuyun 6’* waxes. Relative abundances (%) of individual homologs from each of four common compound classes in the wax mixtures covering the second leaf and the fifth leaf. Bars represent mean ± standard error (n = 4). ^*^p < 0.05; ^**^p < 0.01; ^***^p < 0.001.

Free fatty acids, 1-alkanols, aldehydes and n-alkanes are common wax constituents found from tea leaves as well as many other plant species. The acid fraction was even-numbered homologs, with carbon chain length ranging from C_16_ to C_30_ except C_20._ The second leaf showed maximum abundance of C_28_ and a second maximum at C_26_ (Fig. 5). The fifth leaf waxes contained the same acid homologs except C_22_ and C_30_. C_24_ only showed a trace amount; C_16_, C_18,_ C_26_ and C_28_ exhibited almost even distribution (Fig. 5). The primary alcohol (1-alkanol) fraction contained even-numbered homologs between C_18_ to C_34_ except C_20_ and C_22_. The second leaf exhibited a maximum at C_28_ and C_30_ (Fig. 5). In contrast, C_28_ and C_32_ were enriched in the fifth leaf; C_30_ and C_34_ were absent, and others showed similar abundance (Fig. 5).

The tea leaf waxes were comprised by even-numbered aldehydes ranging from C_26_ to C_30_. The second leaf was dominated by C_30_, followed by C_26_; C_28_ was not detected from the second leaf (Fig. 5). C_30_ is the dominant aldehyde in the fifth leaf, followed by a second maximum at C_28_ (Fig. 5). The alkane fractions in the wax mixtures were featured by odd-numbered homologs from C_21_ to C_35_ except C_23_ and C_33_. C_29_ was the dominant component in the second and the fifth leaf, and other chain length showed similar distribution except C_35_ in the second leaf in which was a second maximum (Fig. 5).

**Figure 6:**
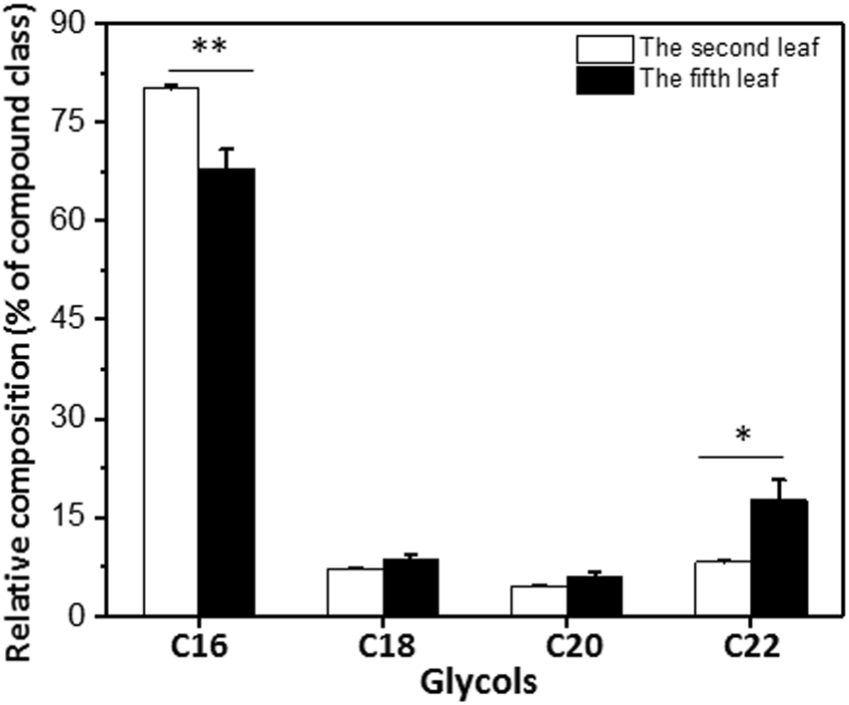
Chain length distributions of glycols in *Camellia sinensis* cv ‘*Fuyun 6’* waxes. Relative abundances (%) of individual homologs from the second leaf and the fifth leaf. Bars represent mean ± standard error (n = 4). ^*^p < 0.05; ^**^p < 0.01; ^***^p < 0.001.

The glycols fraction was even-numbered homologs, ranging from C_16_ to C_22_. The second and the fifth leaf showed maximum abundance of C_16_, and other chain length showed similar distribution level in both leaves (Fig. 6).

### Chain length distributions of wax esters

**Figure 7:**
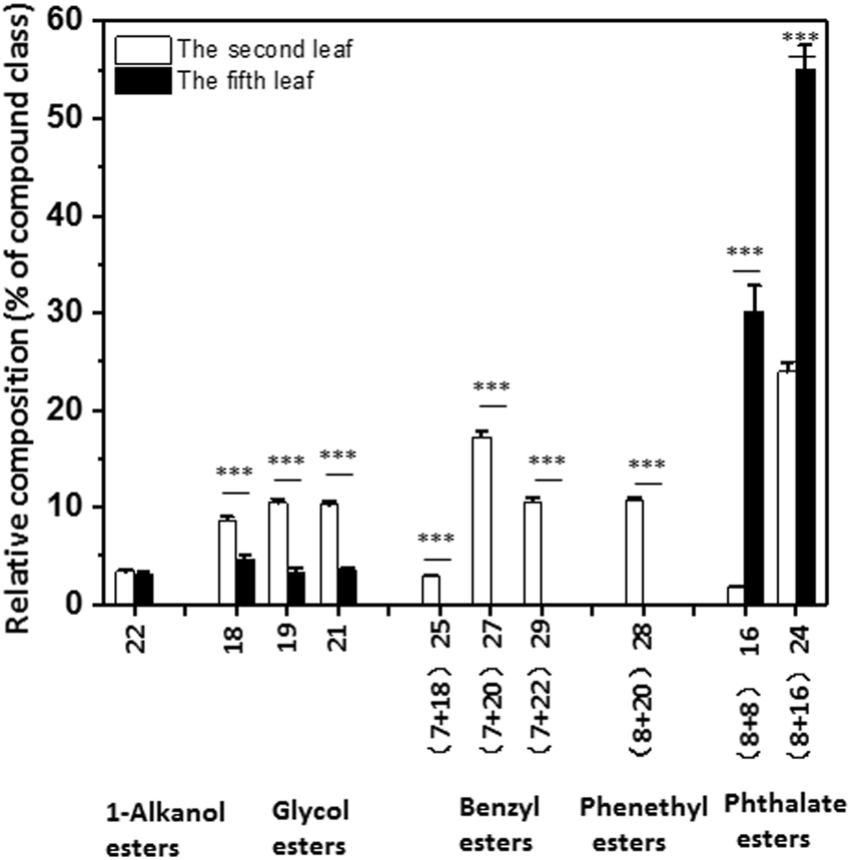
Chain length distributions of esters in *Camellia sinensis* cv ‘*Fuyun 6’* waxes. Relative abundances (%) of individual homologs from the second leaf and the fifth leaf. Bars represent mean ± standard error (n = 4). ^*^p < 0.05; ^**^p < 0.01; ^***^p < 0.001.

Five of the compound classes were found from the second leaf and characterized by ester linkages between fatty acids and various alcohols in extended series of homologs, while only three classes were detected from the fifth leaf (Fig. 7). The 1-alkanol esters were produced from esterification of acetate with primary alcohols, only the C_20_ alkyl chain was detected from the second and fifth leaf. Glycol esters were detected in the second leaf and the fifth leaf with even distribution, and featured by the presence of even-numbered acyl chain C_16_ or C_18_ and short chain alcohols (C_2_) or glycerol (C_3_), with a total carbon number of 18, 19 and 21.

There were two classes of esters formed by long-chain fatty acids and aromatic alcohols. Benzyl esters were formed by esterification of benzyl alcohol with C_18_, C_20_, and C_22_ fatty acids_,_ thus the total carbon numbers were C25, C_27_, and C_29_, respectively. Benzyl esters showed maximum abundance of C_27_, followed by a second maximum of C_29_ in the second leaf. Benzyl esters were undetectable from the fifth leaf. Similarly, phenethyl esters were detected from the second leaf and absent from the fifth leaf, only C_20_ acyl chain length was detected, the total carbon number of phenethyl esters was 28.

Phthalate esters were formed by phthalic acid or terephthalic acid esterification with two short chain alcohols (C_4_ or C_8_) with overall carbon numbers of C_16_ and C_24_. C_24_ showed higher abundance than C_16_ in the second and fifth leaves. Overall, the fifth leaf was dominated by phthalate esters; in contrast, the majority of the ester classes showed more even distribution in the second leaf (Fig. 7).

### The terpenoids composition of tea leaf waxes

**Figure 8:**
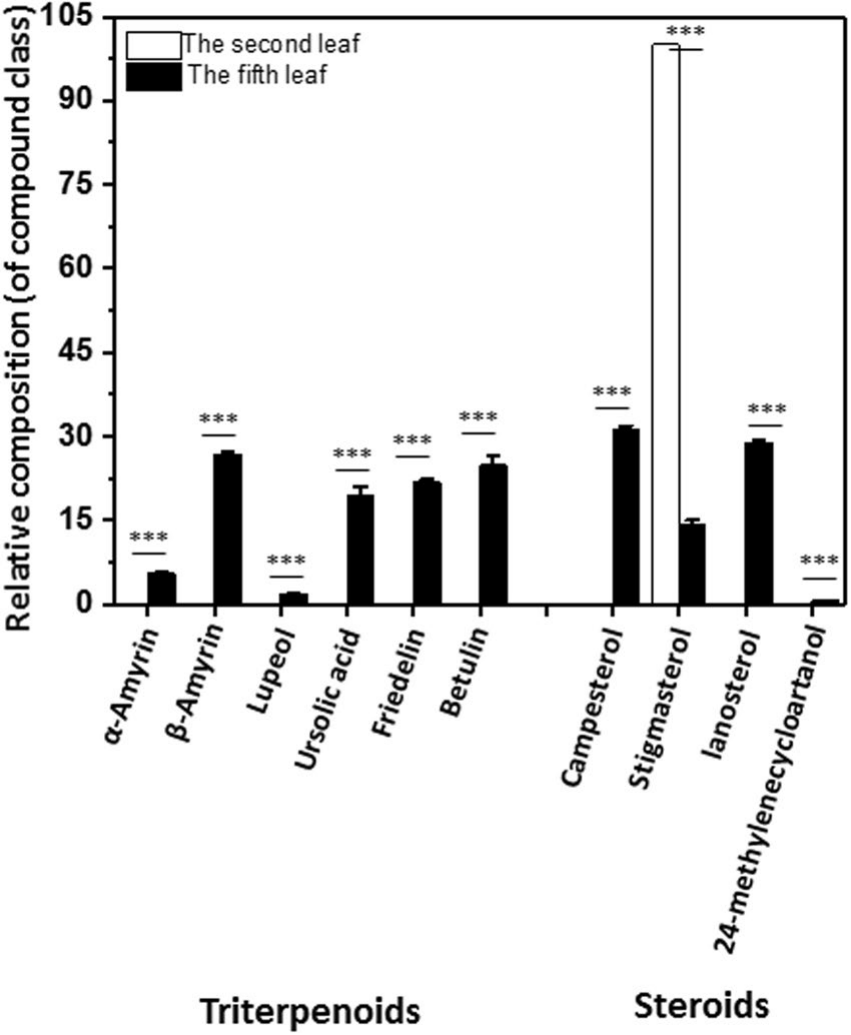
The compound composition of the terpenoids from *Camellia sinensis* cv ‘*Fuyun 6’* in the wax mixtures covering the second leaf and the fifth leaf. Bars represent mean ± standard error (n = 4). ^*^p < 0.05; ^**^p < 0.01; ^***^p < 0.001.

The terpenoids from tea leaf waxes, including triterpenes and sterols, were mainly detected from the fifth leaf, and undetectable from the second leaf except stigmasterol (Figs 4 and 8). Triterpenes were dominant components in the fifth leaf (Fig. 4), include α-amyrin, β-amyrin, lupeol, ursolic acid, friedelin and betulin. β-amyrin and betulin showed maximum abundance, followed by a second maximum for friedelin and ursolic acid; α-amyrin and lupeol were present at low abundance (Fig. 8). Friedelin was identified from tea extracts by NMR^31^, in this study we also identified it from tea leaf waxes (Supplementary Fig. S1).

Four sterols were identified from the fifth leaf wax mixtures, include campesterol, stigmasterl, lanosterol and 24-methylenecycloartanol. Campesterol and lanosterol showed maximum abundance, followed by a second maximum of stigmasterol. Trace amount of 24-methylenecycloartanol was also detected from the fifth leaf (Fig. 8). From the second leaf waxes mixtures only stigmasterol was detected at low amount (Figs 4 and 8).

## Discussion

Cuticle is consisted of a polymeric cutin matrix and soluble cuticular waxes^32^. The epicuticular waxes is deposited on the cutin surface and can be removed by mechanic stripping^33^; in contrast, the intracuticular waxes is embedded within the cutin^34^. It is widely accepted that cuticle is arranged into three layers: the outmost is epicuticular wax film (EW), followed by cuticle proper (C), the cuticular layer is located below cuticle proper. The intracuticular wax layer includes cuticle proper and cuticular layer^30,35^. When the epicuticular waxes were removed from *Arabidopsis* stem by chloroform, the TEM image only showed electron-dense structure, suggesting that the electron-dense structure from TEM image presumably was cutin-rich^24^. In our TEM images (Fig. 2a–d), we observed a slightly electron dense layer just below the cuticle surface, we speculated that could be the cutin proper. Based on the location of cutin proper, the thickness of the epicuticular wax layer and the intracuticular wax layer can be directly measured from TEM images.

It was reported that conventional chemical fixation for TEM sample preparation resulted in the ultrastructure changes of *Arabidopsis* stem cuticle, the authors speculated that the organic solvents (acetone or ethanol) used by TEM sample preparation possibly removed some waxes^24^. The major waxes from *Arabidopsis* stem is alkane, which is more soluble in organic solvents such as chloroform. In contrast, some wax components such as aldehydes and triterpenoids are only slightly soluble in chloroform at ambient temperature^36^. Chloroform is a much stronger wax solvent than acetone or ethanol, it is expected that some wax components (aldehydes or triterpenes) should show very low solubility in acetone or ethanol. Thus, cuticle wax compositions should have dramatic impacts on its solubility in specific organic solvent, it is inappropriate to generalize the solvent effects across plant species. Jetter and Riederer (2016) used enzyme digestion to isolate cuticular membrane from 8 different plant species, then measured cuticle thickness by SEM, they found that the average thickness of the periclinal parts of the cuticles was in the range of 1 µm to 7 µm^17^. Here, we measured cuticle thickness by TEM image, and found that the adaxial cuticle thickness from tea leaf was in the range of 1 µm to 2.5 µm, thus our data was in accordance with that report^17^.

Since new leaves were emerged sequentially after bud break, we first explored the possibility if leaf position on a growing twig could be used as a proxy for leaf developmental stages or developmental status. In this study, two lines of evidences suggested that the second leaf was more likely immature: firstly, the epidermal cell size from the second leaf was similar to the first leaf (Fig. 1g), but leaf area was much larger than the first leaf (Fig. 1h), suggesting that the second leaf still maintained active cell division; secondly, the structural characteristics of the second leaf, including palisad layer formation, mesophyll cell shape, cell packing pattern and intercellular air space, shared higher similarity to the first leaf compared with the third leaf or the fifth leaf (Fig. 1c–f). Although leaf developmental biology has well established the correlation between leaf structural characteristics with their photosynthetic capacity, more direct evidences about its carbon status were required in order for a concrete definition of the second leaf as “immature”. When the twig was left to grow for extended period, the current second leaf gradually moved down by other newly emerged leaves, eventually become fully-expanded and indistinguishable from other fully-expanded leaves. In the tea-making industry, the tender shoots (usually 1 bud with 2 leaves) are harvested to produce tea. Thus, maintaining shoots at tender stage for a longer period is a highly desirable agronomic trait and an intense target for new tea cultivar breeding, since this can elongate the harvest time without compromise of product quality during harvesting season.

Our data indicated that the expansion of epidermal cells attributed to the increase in leaf surface area from the second leaf to the fifth leaf (Fig. 1g,h). Thus, cuticle should also expanded accordingly from the second leaf to the fifth leaf. It still remains an unresolved mystery how cuticle expands with epidermal cell expansion, however, it is reasonable to infer that when the synthesis or transport of specific wax component can’t keep pace with epidermal cell expansion, a dilution effect should occur, this would result in reduced coverage of this specific wax component. The coverage of VLCFA and its derivatives (including acids, 1-alkanols, aldehydes and alkanes) in the second was 3.626 μg/cm^2^; in contrast, the coverage of VLCFA and its derivatives was 1.039 μg/cm^2^ in the fifth leaf (Fig. 4, Supplemental Table S1). Seemingly, the fifth leaf showed lower rate for VLCFA biosynthesis than the second leaf. Considering that the total surface area of the fifth leaf was 3 folds of the second leaf (Fig. 1h), indicating that the total loads of VLCFAs were similar between the second leaf and the fifth leaf. These data likely suggested that VLCFAs and their derivatives were mainly formed in the tender leaf, then these pathways were dramatically turned down with further leaf growth and development. This provided reasonable explanation why green tea leaves were the exceptionally rich plant-sources of policosanol as reported by Choi *et al*.^26^. Our data also indicated that large quantity of policosanol actually was stored in tender leaf cuticular waxes. Primary alcohol is synthesized through acyl-reduction pathway; in contrast, the decarbonylation pathway produces aldehydes, alkanes, ketones and secondary alcohol^37,38^. The dominance of primary alcohol in the second leaf suggested differential regulation of these two pathways in the tender leaf for waxes lipid biosynthesis.

The coverage of triterpenoids and steroids mixture increased dramatically with leaf expansion and growth (Figs 4 and 8). The biosynthesis of triterpenoids and steroids takes place in the cytoplasm through the mevalonic acid (MVA) pathway by using acetyl-CoA as precursor, both pathways share the same intermediate (3S)-2,3-epoxy-2,3-dihydrosqualene. The VLCFAs are synthesized from C16 and C18 fatty acids, leading to the formation of VLCFAs ranging from C24 to C36 in length. VLCFAs synthesis takes place in cytoplasm by fatty acid elongase and uses cytosolic acetyl-CoA as precursor for fatty acid elongation^39^. Thus, the biosynthesis of VLCFAs, triterpenoids and steroids are interconnected at metabolic level; these pathways compete for the same precursor acetyl-CoA from the cytoplasm. From these data we can infer that in the tender leaf VLCFAs pathway is very active, and MVA pathway is not activated; with leaf expansion and maturation MVA pathway is activated, and VLCFAs pathway is turned down, thus more cytosolic acetyl-CoA is fed into MVA pathway for triterpenoids and steroids biosynthesis.

α-amyrin synthase, β-amyrin synthase and lupeol synthase catalyzed the formation of α-amyrin, β-amyrin and lupeol from the same precursor by separate branch pathways^40–42^. The lower amount of lupeol in the fifth leaf suggested relative lower activity of lupeol synthase. α-amyrin synthase and β-amyrin synthase were reported to be multifunctional, such that α-amyrin synthase can synthesize β-amyrin, and β-amyrin synthase can also catalyze α-amyrin production^41^. Thus, a 4- fold higher β-amyrin than α-amyrin in the fifth leaf was unexpected (Figure 8). β-amyrin can be converted into oleanolate which was not detected from the fifth leaf, suggesting that this pathway is not active in the fifth leaf. α-amyrin can be oxidized to ursolic acid which was detected as abundant component, suggesting that most of the α-amyrin was further converted into ursolic acid in the fifth leaf. By adding up the α-amyrin and ursolic acid, the total amount was equivalent to β-amyrin. Thus, our data supported the multifunctional property of α-amyrin synthase and β-amyrin synthase^41^.

There was long speculation that the aroma quality of made tea was positively associated with cuticle wax load, the comprehensive profiling of cuticular waxes from tea leaves in this study offered new insights. The tender leaves (usually 1bud 2 leaves) are plucked to make tea, we found that the cuticular waxes from the second leaf was enriched with fatty acids and their derivatives, which could be further oxidized to produce aroma volatiles during black tea making, including methyl jasmonate, cis-jasmone, hexanal, 3-hexenal, 3-hexen-1-ol^43–47^. Compared with the fifth leaf, the cuticular waxes from the second leaf also were enriched with esters, including benzyl esters, phenethyl esters and phthalate esters. These esters all contain aroma precursor which could be released under the action of enzymes. During black tea making the cell wall was disintegrated during the rolling step, lipases and other enzymes were released from the cell, then could react with aroma precursors during the following fermentation step, thus released the aroma compounds.

In summary, the work herein presented a comprehensive structural and compositional analysis of cuticle from two leaf positions of *Camellia sinensis* cv ‘*Fuyun 6’*, and revealed differential metabolic regulations during tea leaf maturation regarding the cuticular wax biosynthesis. This provides frame work for the functional characterization of related genes in tea tree for wax biosynthesis.

## Materials and Methods

### Plant materials

*Camellia sinensis* cv *Fuyun 6* was grown in a tea garden at Fujian Agriculture and Forestry University. The samples were harvested when rapid growing twigs reached stage of one bud with 6 to7 leaves, the second leaf and the fifth leaf were collected for investigation.

### Transmission electron microscopy

The second leaf and the fifth leaf were taken from same twig, they were 10- and 28-days old after bud break, respectively. Small pieces (2 mm × 4 mm) were taken from the central part of individual leaf, one corner was cut to label the adaxial or abaxial surface. The leaf pieces were transferred into 5% (v/v) glutaraldehyde solution, fixed overnight in 4 °C freezer. In the next day, samples were rinsed three times with 0.1 M PBS buffer (pH 7.2), then post fixed with 1% (w/v) osmium tetroxide in 100 mM PBS buffer at 4 °C freezer for 2–2.5 h. After fixation, samples were rinsed 3 times with PBS buffer for 15 min each: rinsed once with PBS: ddH_2_O (3:1, v/v) for 15 min, once with PBS: ddH_2_O (1:1, v/v) for 10 min, and once with PBS: ddH_2_O (1:3, v/v) for 5 min. They were rinsed once with ddH_2_O, then dehydrated through 30% (v/v) and 50% (v/v) ethanol for 15 min, respectively. Treat samples with saturated uranyl acetate in 70% (v/v) ethanol overnight. In the next day, samples were rinsed with 70% (v/v) ethanol several times to remove unbound uranyl acetate, then dehydrated in 90% (v/v) and 100% ethanol for 17 min, respectively. Repeat the dehydration step in 100% ethanol. Samples were then treated in sequence with acetone: ethanol (1:3, v/v), acetone: ethanol (1:1, v/v), acetone: ethanol (3:1, v/v) and 100% acetone for 17 min, respectively. Repeat the dehydration step again in 100% acetone. Then infiltrated through a graded acetone/Epon/Spurr’s epoxy resin and polymerized at 70 °C for 24 h, the polymerized blocks were sectioned on a Leica Ultracut UCT ultramicrotome. Samples for transmission electron microscopy were sectioned at 70 nm thickness using an ultra 35° diatome diamond knife, the thin sections were collected on 200 mesh copper thin bar grids, and viewed in the HT7700 transmission electron microscope (Hitachi, Japan).

### Light microscopy

Three twigs with same bud break time were labeled, leaves were harvested when the twigs reached stage of 1 bud with 6–7 leaves. Immediately after harvesting, two small pieces (2 mm × 4 mm) were taken from the central part of individual leaf, transferred into 5% (v/v) glutaraldehyde solution, and fixed overnight in 4 °C freezer. The samples were prepared in the same way as TEM samples described above, except that 1.0 µm thickness were sectioned onto glass slides by using ultramicrotome (UC-7RT, Leica, Germany). Samples were stained in 1% (g/v) toluidine blue solution for 15 min, then rinsed three times with distilled water. Slides were observed and photographed under Nikon Ni-U fluorescence microscopy (Nikon, Japan). For cell size determination, 10 to 15 adaxial epidermis cells were measured and averaged.

### Cuticle thickness measurement

Cuticle thickness measurement was based on TEM images. During TEM sample fixation the sample position was carefully adjusted so that cross section was perpendicular to leaf surface, by doing so the inclination effects were minimized. In TEM images cuticle showed whitish appearance. In contrast, epidermis cell wall was stained to dark color, so a clear junction between cuticle and epidermis cell wall was revealed. Cuticle proper formed a slightly electron-dense thin band within the whitish cuticle, thus divided cuticle into epicuticular wax layer and cuticular layer. Three biological replicates were used for cuticle measurement, for each biological replicate at least six measurements were taken from different positions (technical replicates), the results were expressed as average ± standard error (SE).

### Total wax extraction

Total wax extraction follow the method described by Racovita^12^ with minor modification. To exclude potential system contaminations, blank samples were prepared in the same way as other samples, except that no leaves were added. The second leaf and the fifth leaf were removed from twigs, individual leaf was photographed, and leaf area was calculated by Image J software. Twenty of the second leaf were randomly selected and pooled together as one biological replicate; for the fifth leaf 10 leaves were randomly selected and pooled together as one biological replicate. In total, four biological replicates were prepared for the second leaf and the fifth leaf, respectively. Tea leaves were put into glass beaker, 13 mL of chloroform containing 100 µg of internal standard n-tetracosane was added such that the leaves became fully submerged. After stirring for 30 s at room temperature, the chloroform was transferred to another glass vial. The extraction step was repeated once. These two extractions were combined and dried under CentriVap Console (Labconco, KS, USA).

### Derivatization reactions

50 µL N,O-bis(trimethylsilyl)trifluoroacetamide (BSTFA, Aldrich, GC grade) containing 1% trimethylchlorosilane (Aldrich) and 200 µL pyridine (Aldrich, 99.8%, anhydrous) were added to dry wax sample, then heated in a dry bath at 70 °C for 1 hour. Excess reagents were completely removed under a stream of nitrogen gas_,_ the samples were dissolved in 600 µL chloroform for analysis.

### GC-MS and GC-FID analysis

Each sample was divided into two portions for GC-MS (GCMS-QP2010 Ultra, Shimadzu, Japan) and GC-FID (GC-2010 plus, Shimadzu, Japan) analysis, respectively. GC-MS and GC-FID were equipped with same type of capillary GC column (DB-1, 30 m x 0.25 mm × 0.25 µm, Agilent, California, USA). Oven temperature program was set as same for GC-MS and GC-FID: starting at 70 °C, ramp 10 °C min^−1^ to 200 °C, keep constant for 2 min, then ramp 3 °C min^−1^ to 320 °C, stay for 20 min. Helium was used as mobile phase at speed 1.2 mL min^−1^. GC-MS was applied for compound identification. The MS detector setting was: EI-70eV, ionization source temperature 230 °C. FID detector was used for quantification of individual wax homologs based on normalization of peak areas against that of the internal standard.

### Data analysis

Data analysis was performed by using Microsoft Excel 2016 and Origin2017 software, data was expressed as mean ± standard error (SE).

## Electronic supplementary material

Supplementary Table

Supplementary Figures
