# Supplementary material for: Tender leaf and fully-expanded leaf exhibited distinct cuticle structure and wax lipid composition in Camellia sinensis cv Fuyun 6
Source: Sci Rep. 2018 Oct 8;8:14944. doi: 10.1038/s41598-018-33344-8 (PMC6175935; doi:10.1038/s41598-018-33344-8)

Tender leaf and fully expanded leaf exhibited distinct cuticle structure and wax lipid composition in *Camellia sinensis cv Fuyun 6*

Xiaofang Zhu[1,2,3], Yi Zhang[3], Zhenghua Du[3], Xiaobing Chen[3], Xin Zhou[3], Xiangrui Kong[2], Weijiang Sun[4], Zijian Chen[5], Changsong Chen[2*], Mingjie Chen[3*]

[1]College of Horticulture and Fujian Provincial Key Laboratory of Haixia Applied Systems Biology, Fujian Agriculture and Forestry University, Fuzhou, Fujian 350002, China
[2] Tea Research Institute, Fujian Academy of Agricultural Sciences, Fuan, Fujian 355000, China
[3] FAFU-UCR Joint Center/Horticultural Plant Biology and Metabolomics Center, Haixia Institute of Science and Technology, Fujian Agriculture and Forestry University, Fuzhou, Fujian 350002, China
[4]Anxi College of Tea Science, Fujian Agriculture and Forestry University, Fuzhou, Fujian 350002, China
[5] Engineer School, University of Missouri, Columbia, Missouri 65211, USA
*Correspondence authors:
Email: chenmj@fafu.edu.cn； ccs6536597@163.com

Supplementary information

Supplementary Figure S1. GC-MS identification of Friedelin.

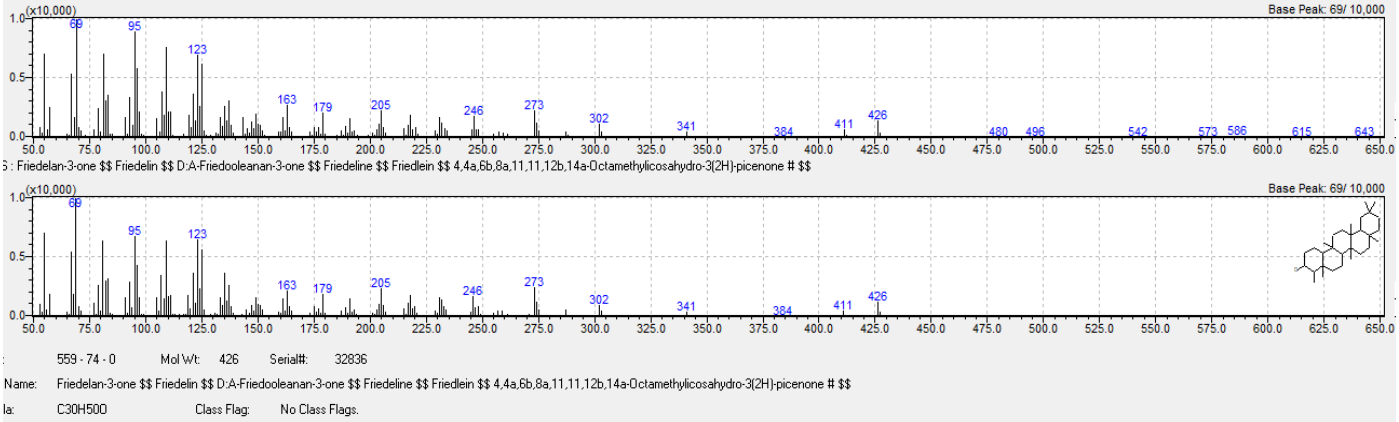

Supplementary Figure S2. GC-MS identification of 1-alkanol ester.

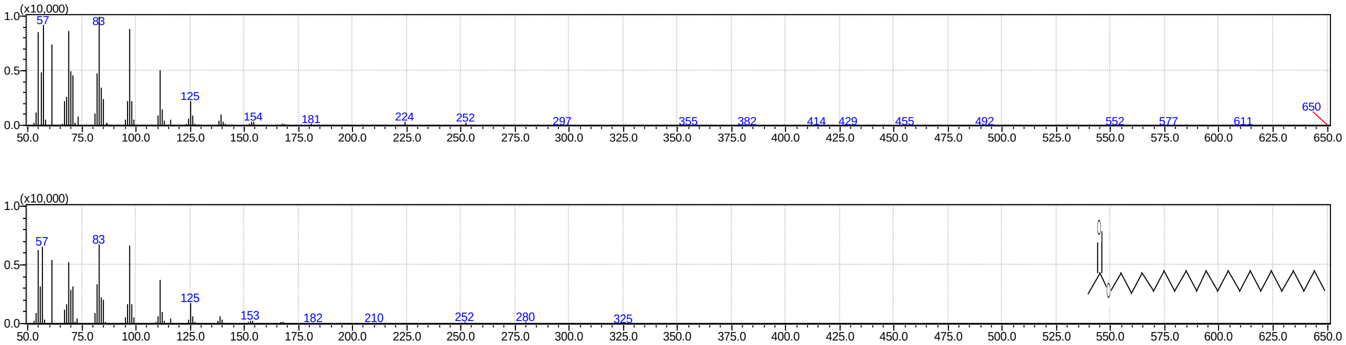

Supplementary Figure S3. GC-MS identification of 4,4-dimethylzymosterol. Another 2 top candidates also were shown at bottom for comparison.

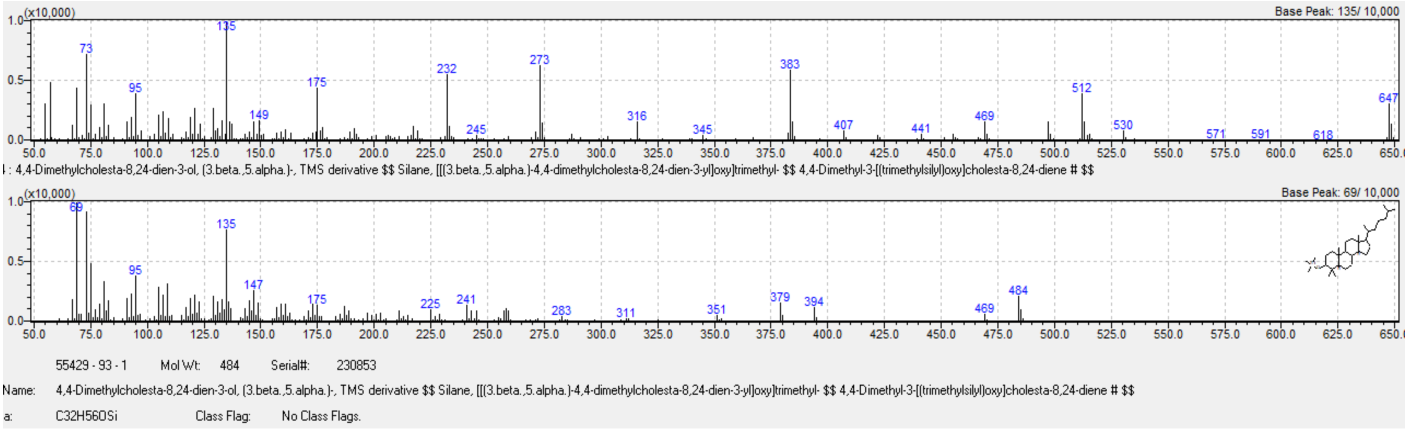

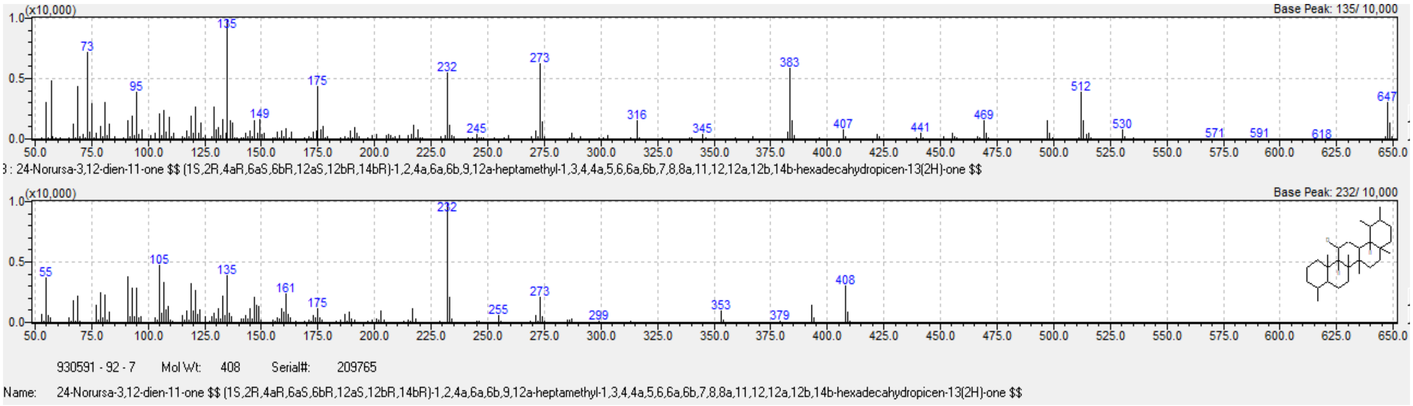

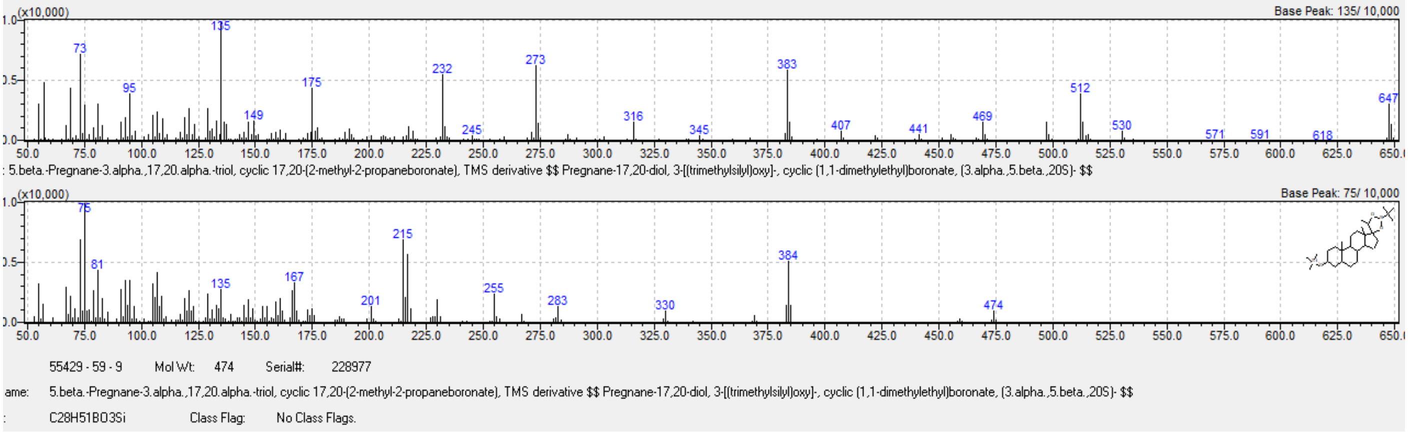

Supplementary Figure S4. GC-MS identification of ketone.

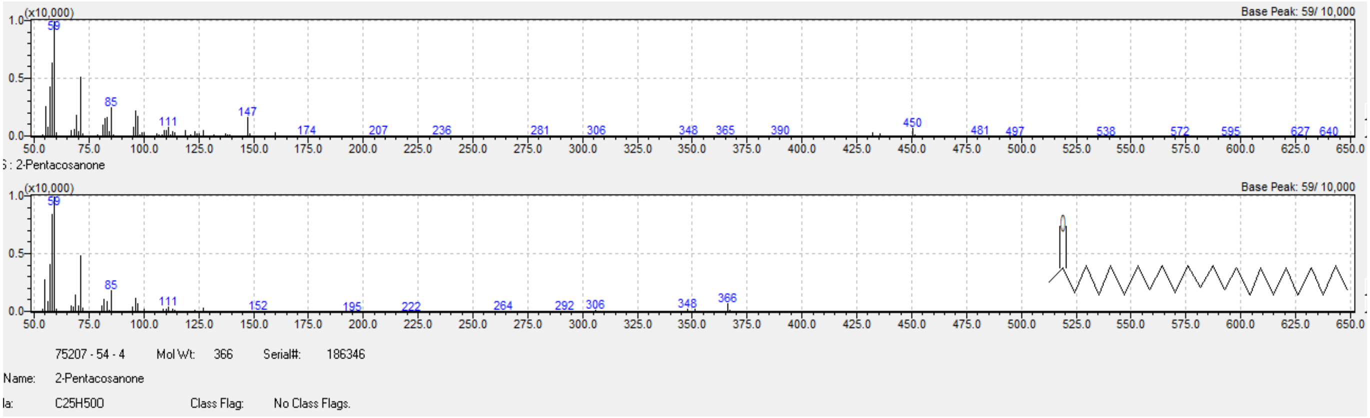

Supplementary Figure S5. GC-MS identification of benzyl esters-C25, C27 and C29.

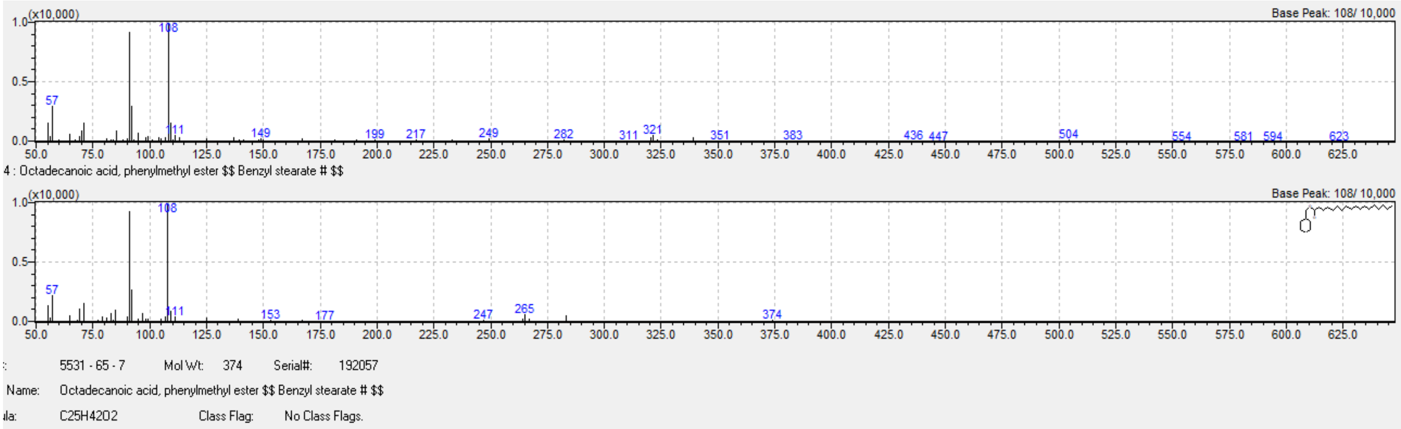

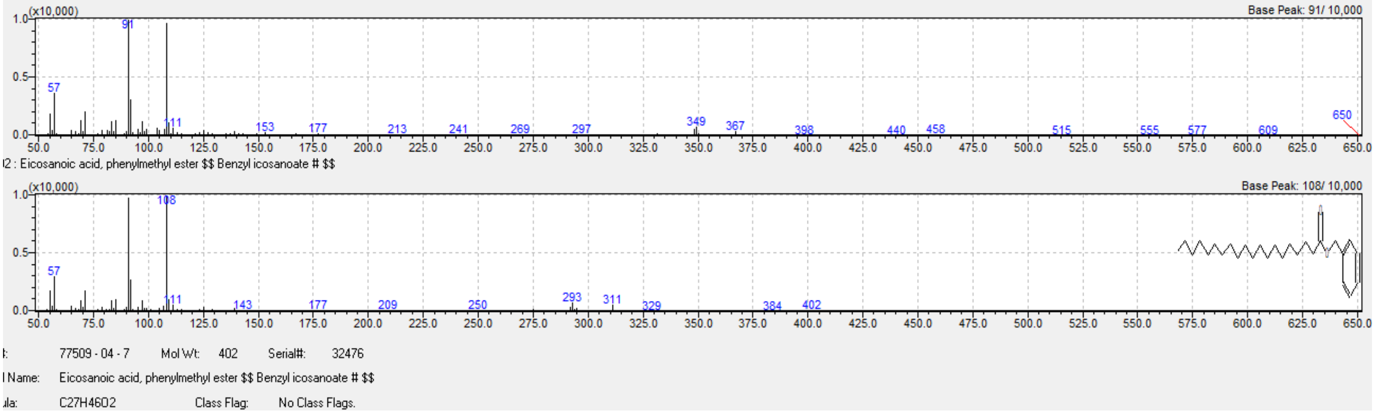

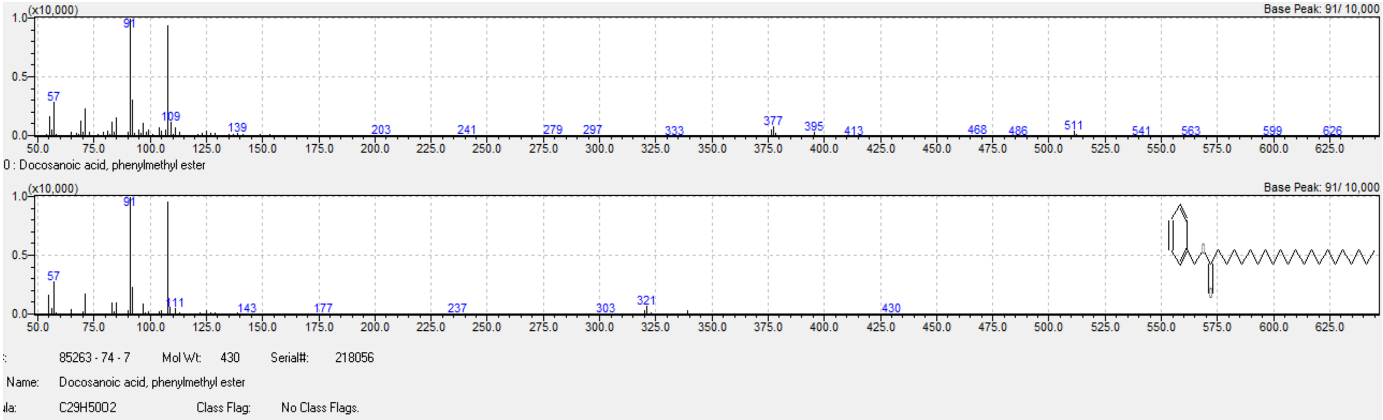

Supplementary Figure S6. GC-MS identification of phenethyl esters-C28.

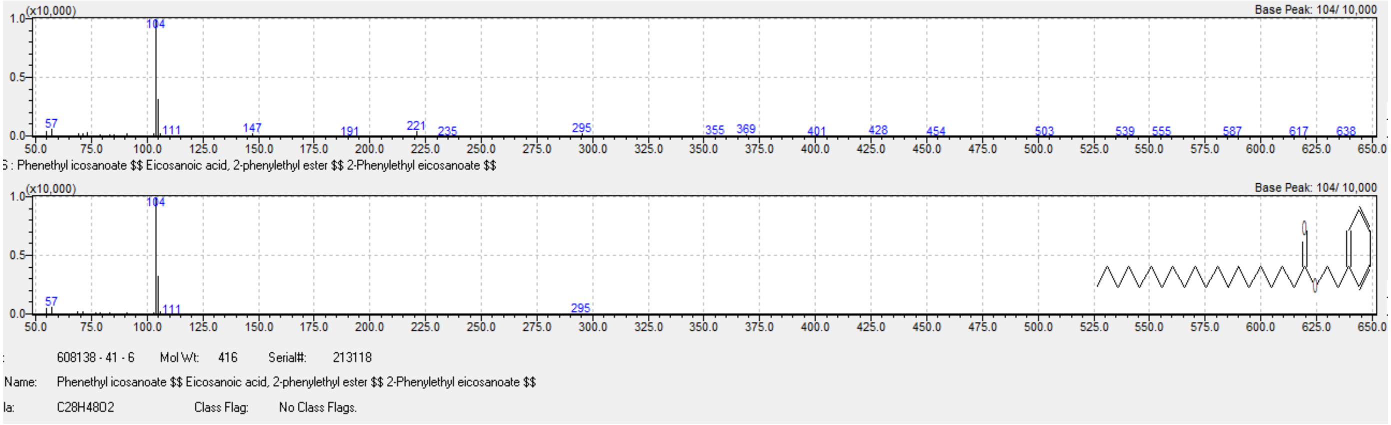

Supplementary Figure S7. Overlay of chromatography from blank sample and wax sample isolated from the second leaf. Black line: blank sample; red line: wax isolated from the second leaf.

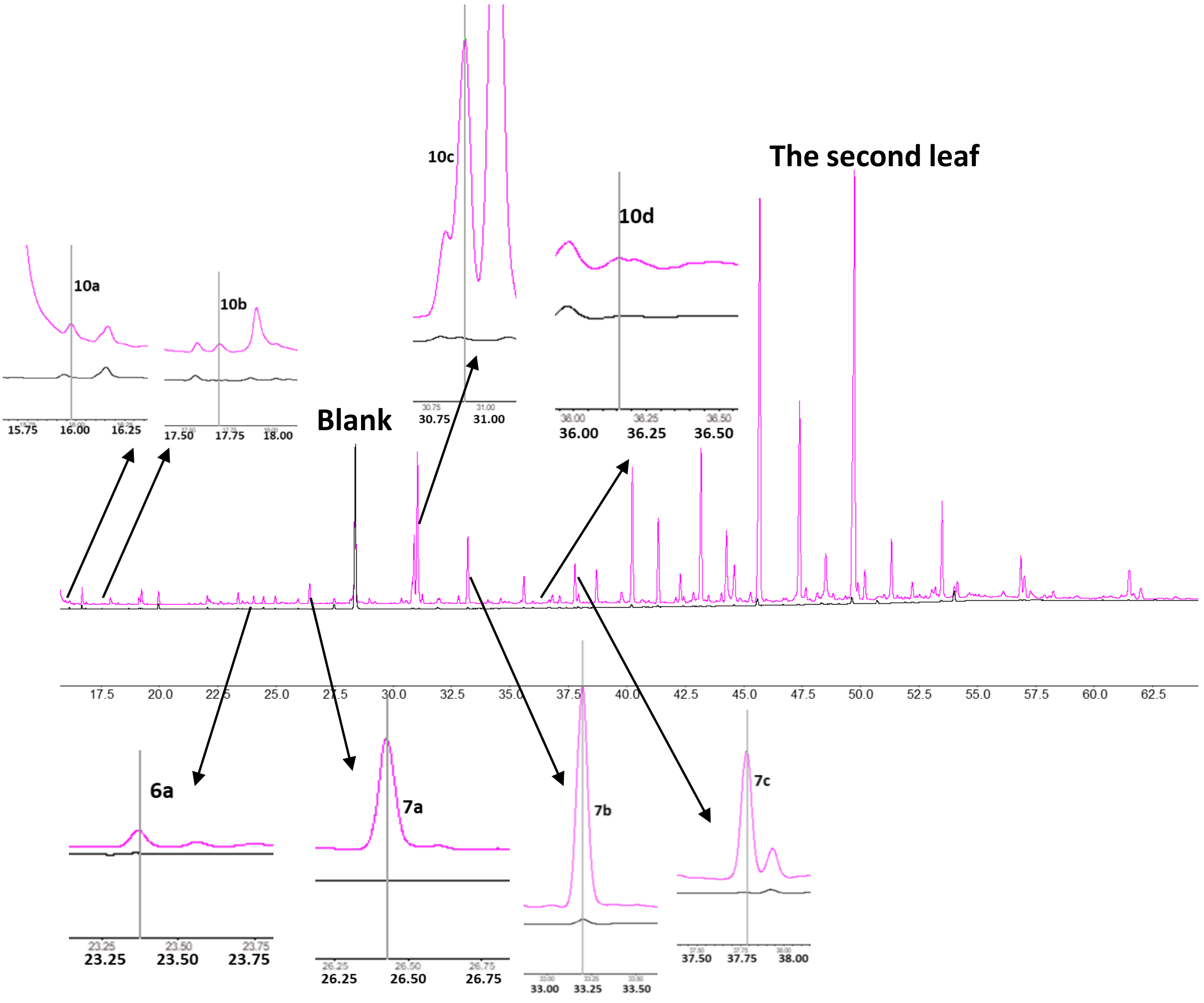

Supplement: Supplementary file 2 — Supplementary Figures [file 41598_2018_33344_MOESM2_ESM.pdf]
